# Children’s Health Habits and COVID-19 Lockdown in Catalonia: Implications for Obesity and Non-Communicable Diseases

**DOI:** 10.3390/nu13051657

**Published:** 2021-05-13

**Authors:** Paula Sol Ventura, Ana F. Ortigoza, Yanira Castillo, Zelmira Bosch, Sara Casals, Cristina Girbau, Jose M. Siurana, Amalia Arce, Marisa Torres, Francisco J. Herrero

**Affiliations:** 1 Department of Pediatric Endocrinology, Hospital HM Nens, HM Hospitales, 08009 Barcelona, Spain; zelmira.bosch@gmail.com (Z.B.); mtorres@mail.hmhospitales.com (M.T.); jherreroespinet@gmail.com (F.J.H.); 2 Fundació Institut d’Investigació en Ciències de la Salut Germans Trias i Pujol (IGTP), 08916 Badalona, Spain; 3 Urban Health Collaborative, Drexel University, 3600 Market Street, Philadelphia, PA 19104, USA; afo25@drexel.edu; 4 Pediatrics Department, Hospital HM Nens, HM Hospitales, 08009 Barcelona, Spain; yaniracastillo12@gmail.com; 5 Nutrition Department, Hospital HM Nens, HM Hospitales, 08009 Barcelona, Spain; sara_casals@hotmail.com (S.C.); cgirbau@hmhospitales.com (C.G.); 6 Cardiology Department, Hospital HM Nens, HM Hospitales, 08009 Barcelona, Spain; jmsiurana@hotmail.com; 7 Autonomous University of Barcelona, 08193 Bellaterra, Spain; 8 e-Salud Department, Hospital HM Nens, HM Hospitales, 08009 Barcelona, Spain; amalia.arce.casas@gmail.com

**Keywords:** children, adolescents, Mediterranean diet, physical activity, sleep disorder, COVID-19, non-communicable disease, risk factors, sedentarism

## Abstract

Lockdown during the COVID-19 pandemic imposed changes in children’s daily routine that could lead to changes in behavior patterns. Using a survey targeted at children under 17 years of age, we described dietary (adherence to Mediterranean diet, AMD) and sleeping habits (disorders of initiating and maintaining sleep) after the implementation of lockdown, and examined the probability of the inadequate frequency of physical activity (PA) and use of TV and electronic devices (TV-ED) before and after lockdown through generalized estimating equation models, accounting for age and gender differences. From 3464 children included, 53.2% showed optimal AMD; 79.2% referred to delayed bedtime; and 16.3% were suspected of sleeping disorders after the implementation of lockdown. Delay in bedtime was more frequent among children older than 6 years, and inadequate sleeping hours among those younger than 11 years. There were no gender differences in AMD or sleeping habits. The odds of inadequate frequency of PA and TV-ED use were greater after lockdown, with a greater risk for TV-ED use. Boys were at greater risk of inadequate PA frequency and TV-ED use. Odds ratio of inadequate PA was greater at older ages. Lockdown could influence changes in children’s habits that could lead to risk factors for non-communicable diseases during adulthood if such behaviors are sustained over time.

## 1. Introduction

Since the early stages of the COVID-19 pandemic, countries have implemented different strategies to slow down the spread of viral transmission [1]. In Spain, mitigation strategies led to a nationwide lockdown from 15 March to 21 June, 2020. Lockdown measures implied that all residents remained at home, leaving only for essential needs such as buying food and medicines, seeking medical care, or attending essential jobs [2]. Schools and universities were encouraged to continue through online classes [2]. Children younger than 14 were unable to leave their houses unless their caretaker needed to carry them during a permitted activity [2].

Measures to contain COVID-19 represented a sudden and radical change in children’s routines that could impact on their individual habits and behaviors [3]. The impact of lockdown on healthy habits is yet hard to predict. For example, adherence to Mediterranean diet (AMD) could be, on one hand, reduced due to limited access to fresh food and due to the compulsive eating of sugar-rich food because of the boredom and anxiety derived from the lockdown. On the other hand, less exposure to fast food stores and more time for preparing meals at home could favor the implementation of a healthy diet during the lockdown.

The loss of usual work and school schedules, as well as the anxiety and stress generated by COVID-19-related information, could also adversely affect children’s sleeping routines. Additionally, the practice of physical activity (PA) among children could be decreased due to time and space limitations and the lack of social stimuli related to its practice [4]. The increased exposure to television and electronic devices (TV-ED), as a means of entertainment and education, could increase the time children spent on sedentary activities. Furthermore, setting limits on children’s sedentary behaviors could be difficult in conditions of household stress.

The potential changes in children’s habits derived from lockdown are of concern among pediatricians and public health practitioners, as many of these changes in lifestyle are difficult to reverse once they are adopted [5], and could represent an increasing risk for obesity and non-communicable disease during adulthood if they are sustained over time [6]. It becomes important to examine the changes that lockdown measures impose on children’s habits in order to design and implement public health strategies that could diminish the loss of healthy habits among children. 

The objective of this study is to describe dietary and sleeping habits of children three to five weeks after the implementation of lockdown in Catalonia, Spain, and to examine changes in the frequency of PA and use of TV-ED before and after the implementation of lockdown.

## 2. Materials and Methods

We retrospectively retrieved information on children’s behavior before and three to five weeks after the implementation of lockdown in an uncontrolled study group. We included children younger than 17 years who lived in Catalonia during the nationwide implementation of lockdown in Spain.

An online structured survey in Spanish was distributed through a convenience sampling strategy between 7–18 April 2020 to parents or responsible caretakers through networks of student parents’ associations (‘Asociaciones de madres y padres de alumnus’, AMPA) and social media platforms. Informed consent was obtained from responsible adults who voluntarily and anonymously answered the questionnaire on behalf of all children living in their household. Each respondent was also encouraged to send the survey to other potential participants in their network. At least five AMPA from private and public institutions were contacted during the recruitment process (~400 students each). Details on the survey questionnaire are included in Supplementary Materials.

We received information from 4730 children across Spain, from which 26 questionnaires were discarded as duplicates. We only included children living in the Catalonia region in this study (*n* = 3464), which includes the provinces of Barcelona, Girona, Tarragona, and Lleida. Children living in other regions of Spain (*n* = 1235) or with missing information on their province of residence (*n* = 5) were not included in the analysis. The overall response rate was 73%. Characteristics of children included and excluded from this study are shown in Supplementary Table S1. 

### 2.1. Data Collection

The survey questionnaire was designed by pediatricians from Hospital HM Nens, HM Hospitales, and approved by the IRB Committee. The questionnaire consisted of three sections adapted from standardized questionnaires that aimed to examine: (i) adherence to Mediterranean diet (AMD) after the implementation of lockdown; (ii) sleeping habits after the implementation of lockdown; and (iii) PA frequency and time spent in front of screens (TV or any other electronic devices) before and after the implementation of lockdown. Information on AMD and sleeping habits were only asked after the implementation of lockdown to reduce recall bias and to avoid an extensive questionnaire [7]. Information previous to the lockdown was asked as the usual habit of the child during a ‘normal’ week or day in child’s life. As many families and children could have change habits even several days or weeks before the implementation of lockdown due to fear or concerns about the viral spread, we avoided specifying any number of days/weeks before lockdown in the wording of the questions on usual routines.

We also collected demographic characteristics of children such as gender, age range, province of residence, and the number of days after lockdown children had spent by the time of responding to the survey.

### 2.2. Adherence to Mediterranean Diet

Adherence to Mediterranean diet (AMD) was assessed through the Mediterranean diet quality index for children and adolescents (KIDMED score). Details on how the score was calculated are described elsewhere [8]. KIDMED scores range from −4 to 12 points, where higher values denote higher AMD. Score values can then be categorized into: (i) high or optimum AMD (KIDMED 8 to 12); (ii) medium AMD (KIDMED 4 to 7); and (iii) low AMD (KIDMED equal or less than 3 points). In our study, we considered both KIDMED score and categories of AMD for describing dietary habits after the implementation of lockdown.

### 2.3. Sleeping Habits

Our survey included a set of questions for detecting disorders of initiating and maintaining sleep (DIMS) extracted from the sleep disturbance scale for children (SDSC) [9]. Characteristics of the SDSC are detailed elsewhere [9]. The DIMS assessment independently evaluates sleeping disorders related to bedtime resistance, night awakenings, sleep duration, and sleep latency. Scores range from 7 to 35, with values above 16 suggesting disorders of initiating and maintaining sleep. The item in the DIMS domain assessing sleeping hours (“How many hours of sleep does your child get on most nights”) was used also as a separate question. Answers to this question were then categorized into ‘adequate’ (≥9 h/night for children <11 years, and ≥8 h/night for children ≥11 years) or ‘inadequate’ sleep [10]. Additionally, we included a question assessing the delay in bedtime after lockdown (“After lockdown, does your child go to sleep later than prior to lockdown?”).

### 2.4. Physical Activity

PA was assessed with two questions that compared after-school PA before lockdown and leisure PA after the implementation of lockdown (“Under normal conditions (without lockdown), how many days a week does your child engage in physical activity outside of school?”; “During lockdown, how many days a week does your child exercise between 30 and 60 min a day?”). These questions were designed based on WHO recommendations, and we considered adequate frequency of PA when children performed PA for one or more hours per day, for at least 5 days in a week [11]. Three additional questions assessed whether leisure PA after lockdown was performed with relatives or alone (“During lockdown, how many days a week does your child exercise alone?” and “During lockdown, how many days a week does your child exercise with other family members?”), and the kind of leisure PA performed after lockdown (“Indicate the sport (s) that your child does during lockdown”).

### 2.5. Time Spent on TV and Electronic Devices

Two questions compared the number of days children spent more than two hours/day either watching TV or using electronic devices for entertainment purposes in a week, before and after the lockdown (“Under normal conditions (without lockdown), how many days a week does your child use television, computer (not academic) or mobile for more than 2 h a day?”; “During lockdown, how many days a week does your child use television, computer (not academic) or mobile phone for more than 2 h in a day”). We considered inadequate frequency in use of TV-ED when children spent more than 2 h per day either watching TV or using electronic devices for more than 2 days a week [11].

### 2.6. Statistical Analysis

We described demographic characteristics of participants, and AMD and sleeping habits after the implementation of lockdown by gender and age range. We also showed changes in the frequency of PA and TV-ED use before and after the implementation of lockdown in the overall study population.

We examined changes in the odds of inadequate frequency of PA and TV-ED use among children before and after the lockdown by using generalized estimating equation models (GEE) with empirical standard errors. GEE allows estimating the population-averaged probability of inadequate frequency of PA and TV-ED use, while accounting for within-children correlations across measures over time [12]. We also examined whether outcome differences varied by gender and age range by testing interactions. We used Statistical Package for Social Science SPSS 23.0 (IBM Corp., Armonk, NY, USA) for descriptive analysis and SAS9.4 (SAS Institute Inc., Cary, NC, USA) for the GEE models.

## 3. Results

A total of 3464 children were included, of whom 37.3% were under 6 years of age, and 36.5% and 26.2% were between 6–10 and 11–16 years, respectively (Table 1). Boys and girls were almost equally distributed. The average amount of days that children stayed at home by the time of the survey was 26.8 days. Most of participants (90.2%) were from the Barcelona province (Table 1).

Based on responses provided by parents, the mean KIDMED score in the overall population was 7.5 (Table 2), and the score was significantly higher among children under 6 years of age (Table 3), with no differences among boys and girls (Table 2). Approximately half of the participants (53.2%) showed optimal AMD, with no differences across gender (Table 2). Optimal AMD was higher among children under 6 years of age (Table 3). A summary of the responses to KIDMED questionnaire is presented in Supplementary Table S2.

After the implementation of lockdown, most children aged 6–10 and 11–16 showed a delay in their bedtime (84% and 88.2%, respectively) (Table 3). Parents reported that almost 20% of the children showed inadequate hours of sleep, being it more frequent among children aged 6–10 (28.5%), without differences by gender (Table 3). Children under 6 years of age had a higher DIMS score and were more frequently suspected of disorders in initiating and maintaining sleep (Table 3).

**Figure 1:**
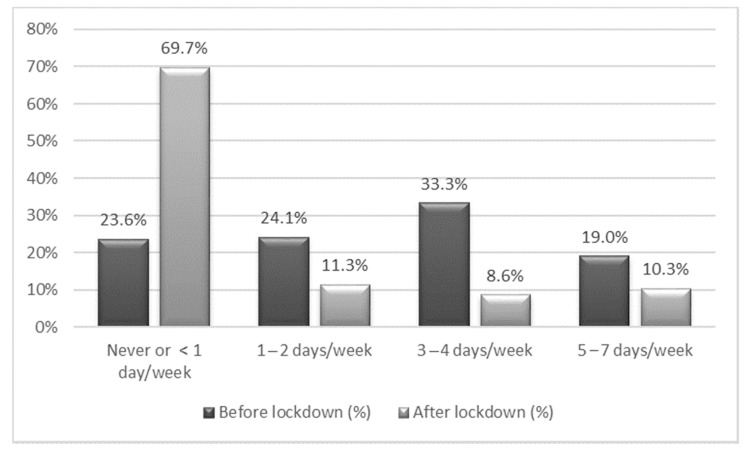
Percentage of children performing more than one hour of physical activity per day, before and after the lockdown. Each column represents the percentage of children in the sample that performed one hour of physical activity per day, before (dark gray) and after (light gray) the lockdown, by weekly frequency.

Before lockdown, more than 50% of children did PA after school for at least one hour per day for three or more days per week (Figure 1), and 19% of children met the requirements for adequate PA (at least one hour of PA per day for more than 4 days per week). After the implementation of lockdown, almost 70% of children reported not doing a minimum of an hour of PA on any day of the week, and only 10.3% reported an adequate frequency of PA (Figure 1). Characteristics and mode of PA performed after lockdown based on parent’s responses are described in Supplementary Figures S1–S4.

**Figure 2:**
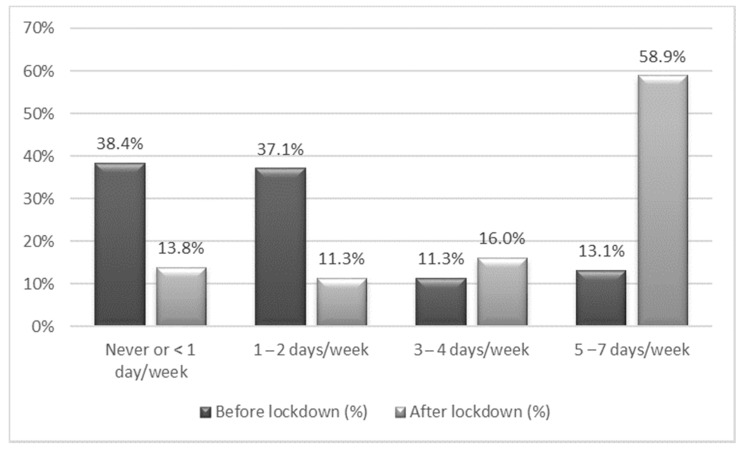
Percentage of children spending more than two hours using non-academic use of TV or electronic devices (TV-ED) per day, before and after the lockdown. Each column represents the percentage of children in the sample that spent more than two hours using TV-ED per day, before (dark gray) and after (light gray) the lockdown, by weekly frequency.

Before lockdown, almost 75% of children used TV-ED (non-academic) for more than two hours on less than three days a week, approaching to the recommendations of adequate frequency of TV-ED use (Figure 2). After lockdown, around 75% of children used TV-ED for more than two hours on three or more days per week (Figure 2). A third of participants performed PA guided by screens, given the information provided by parents (Supplementary Figure S4).

The odds of inadequate frequency of PA after lockdown was twice as likely compared to the odds before lockdown (OR 2.0 95% CI 1.8 to 2.6). The odds ratio of inadequate frequency of PA was greater among boys, and it increased with age for both gender (Table 4). Among boys aged 11–16, for example, the odds of the inadequate frequency of PA after lockdown was five times as likely, as compared to before lockdown (OR 5.0 95% CI 3.3 to 7.6), while among girls of same age, the odds of the inadequate frequency of PA after lockdown was 2.4 times as likely, as compared to before lockdown (OR 2.4 95% CI 1.7 to 3.4) (Table 4).

In the overall sample, the odds of the inadequate frequency of TV-ED use after lockdown was nine times as likely, as compared to before lockdown (OR 9.2 95% CI 8.4 to 10.1). The OR of the inadequate frequency of TV-ED use was greater among boys (Table 5) and among children aged 6–10.

Sensitivity analysis restricting the sample to singleton children did not show significant differences in the OR of either the inadequate frequency of PA or in the use of TV-ED.

## 4. Discussion

Our study showed that AMD was optimal in half of the study population, with worse adherence among children older than 6 years of age. Delay in bedtime was frequent, especially among children older than 6 years of age, while inadequate sleeping hours were more frequent among children aged 6–10 years. DIMS were more likely among children under 6 years of age. No differences between gender were found in AMD or sleeping habits. Boys showed a greater risk of the inadequate frequency of PA and use of TV-ED after lockdown. The probability of inadequate PA frequency was greater for older ages, while the probability of the inadequate frequency of TV-ED use was greater among children from ages 6 to 10 for both genders.

The proportion of optimal AMD in our population (53%) was quite similar as the described before the COVID pandemic in children from Spain in 2004 (46% nationwide and 52% in the northeast region, which includes Catalonia) [8]. It is possible that AMD was not highly impacted by the lockdown due to different counteracting effects derived from it. On one hand, the restrictions imposed by leaving home only for permitted activities could result in limited access to fruits and vegetables [13]. However, remaining at home and closure of non-essential businesses could have reduced the exposure to processed food such as fast-food restaurants (Supplementary Table S2).

Regarding sleeping habits, we found that more than 80% of children aged 6–16 reported a delay in their bedtime during lockdown, as compared to their usual bedtime before the lockdown. Similar results among preschool children in China showed changes in sleep patterns characterized by later bedtimes [14]. They also found in the population studied later wake times, longer nocturnal periods, and shorter nap sleep durations during lockdown, as compared to a previous sleep assessment in 2018 [14]. Changes in children’s usual day routine during lockdown could have impacted their sleeping habits in a similar way to how shift patterns occur during school breaks [15,16]. Delay in bedtime, as well as inadequate sleeping hours, could be, in part, triggered by an increased exposure to screens, as nighttime exposure to bright light suppresses melatonin production [17]. In our study, inadequate sleeping hours were more frequent among children aged 6–10 years, which was also the group with the greater increase in screen exposure after lockdown. We noted that the proportions of sleeping disorders reported in our study were consistent with the ones found in children before the COVID-19 pandemic in Spain. In a study that examined the sleeping habits of children aged 2–14 during the period from 1987 to 2011, De Ruiter et al. found that almost 45% of children in Spain did not meet the recommended sleeping hours [18]. Cassanello et al. also found that more than 30% of parents of children aged 3–36 months perceived that their children had short night sleep hours and longer periods of nocturnal awakenings [19]. The greater proportion of children under 6 years with sleep disturbances after lockdown could be explained, in part, by the high dependence that small children have to their family environment [20]. In a context of greater familial stress and changes to household routines due to lockdown, stress among children can be also manifested as sleeping disorders [21,22].

Our study showed significant differences in the frequency of PA and the use of TV-ED before and after lockdown. The probability of the inadequate frequency of PA (performing one hour or more of PA per day, for less than 5 days in a week) in the overall study population was almost doubled after lockdown, while the probability of inadequate frequency in TV-ED use (spending more than 2 h per day either watching TV or using electronic devices for more than 2 days a week) was nine times higher after lockdown. Similar patterns have been described in the USA during the pandemic, where parents perceived significant decreases in their children’s PA and significant increases in sedentary behavior during the first months of the COVID-19 lockdown as compared to the pre-COVID period (February 2020) [23]. We also observed that gender differences in the probability of the inadequate frequency of PA increased with age. The age differences described in our study are consistent with the findings in the U.S.A. during lockdown, where older children (9–13 years) were reported as spending more time playing computer or video games, or using electronic devices for leisure compared to younger children (5–8 years) [23].

Constraints in access to outdoor places after lockdown may have affected the duration and frequency of leisure PA, particularly among boys, as they usually practice outdoors sports [23], while girls are, in general, more engaged in activities such as dancing and workout training, which could still be performed indoors after lockdown. Outdoor constraints may also have a greater influence at older ages because gendered patterns of leisure activities are more marked towards adolescence. We noticed that, in our study, many children reporting a high frequency of PA after lockdown were those that performed activities with other relatives in the household. Adult guidance and engagement during children’s PA could be a valuable strategy for maintaining adequate levels of PA after lockdown (Supplementary Table S3) [24].

Differences in the inadequate frequency of TV-ED use before and after lockdown were greater than the differences seen for PA. This could be partly explained by the fact that PA during lockdown could have been guided through electronic devices, resulting in a relative increment of screen time with respect to active time [25]. The fact that boys are more likely to engage in video games for leisure [26], which could have been increased during lockdown due to a lack of daily routines that structure their use of time, could also explain the higher risk of the inadequate frequency of TV-ED use seen in boys compared to girls [27].

Results from this study show that mitigation measures during the COVID-19 pandemic could lead to the acquisition of behavioral patterns related to obesity and non-communicable disease (NCD) risk factors such as sleep disturbance and sedentarism [17,28]. These habits could potentially persist beyond the duration of the lockdown, worsening the pre-existing obesity pandemic that was already affecting children in middle- and high-income countries [16,27]. Therefore, it is necessary that these potential long-term consequences are considered in order to establish timely strategies that could diminish the development of non-communicable disease risk factors among children.

Establishing daily routines, with scheduled activities and limited screen exposure time during lockdown could play a key role in maintaining good sleeping and dietary patterns. Allowing outdoor activity, even for small groups, could be beneficial for maintaining adequate levels of PA and for coping with stress through social interactions.

Even when other studies have assessed health behavior among children during the COVID-19 pandemic in Spain [13], ours is, to our knowledge, the largest survey carried out in Catalonia assessing the differences in PA and TV-ED use before and after lockdown, and dietary and sleep habits after lockdown with standardized questionnaires.

Although the uncontrolled design limits the possibility of establishing the true impact of lockdown measures on children’s health behavior, the assessment of outcomes before and shortly after the lockdown reduced the influence of temporal trends and recall bias that usually confound uncontrolled before/after studies. We noticed, however, a limitation in the way information was retrieved, as parents reporting children’s behavior could not only be plausible of recall bias, but also of certain inaccuracy, given that parental perceptions of child behaviors may not be the same as the perceptions children have of their own behaviors [29]. Nevertheless, it has been established that parental information is a method frequently used among scholars examining behavioral and developmental problems among young children, particularly in the study of sleeping habits [30] (References [31,32,33,34,35,36,37] are cited in the Supplementary Materials).

Our methodological approach allowed us to account for unmeasured within-children characteristics that remain invariant over time such as individual or family socioeconomic status. Although our sample included children from different socioeconomic backgrounds attending public and private schools, it is possible that more disadvantaged socioeconomic groups with no internet access and not attending schools may have been underrepresented in our sample.

In this study we were not able to include an objective measure of the changes in children’s weight before and after the lockdown. However, we were able to register whether caregivers perceived any weight gain in children. We found that almost 25% of children included in the sample appeared to show weight gain, this being more frequent among those children with suboptimal (low and medium) adherence to Mediterranean diet (Table S4).

Our findings contribute to an initial approach in understanding the changes that lockdown measures could have on children’s health, and on the development of health risk factors over the life course.

Further studies will need to assess whether the risk of inadequate frequency of PA and TV-ED use examined in this study during the first weeks of lockdown changes over time, in order to have a better knowledge of the long-term consequences that lockdown measures could have on children’s health behaviors.

## 5. Conclusions

Our study showed higher levels of sedentarism and lower levels of physical activity among children and adolescents after the implementation of lockdown as compared to in their previous lifestyle. This change in behavioral patterns at an early age pose a public health concern, as it could lead to risk factors for obesity and cardiovascular disease during adulthood if such behaviors are sustained over time.

It is necessary that the potential long-term consequences of COVID-19 mitigation measures are considered by decision-makers and public health stakeholders in order to design and implement timely strategies that could diminish the impact that lockdown may have on children’s lifestyle.

## Figures and Tables

**Table 1:** Demographic characteristics of participants by gender.

	Total *n* = 3464	Girls *n* = 1656 (47.8%)	Boys *n* = 1727 (49.9%)	Missing Gender *n* = 81 (2.3%)
Age range, *n* (col %)				
<6 years	1291 (37.3)	618 (37.3)	645 (37.4)	28 (34.6)
6–10 years	1263 (36.5)	603 (36.4)	632 (36.6)	28 (34.6)
11–16 years	910 (26.2)	435 (26.3)	450 (26.1)	25 (30.9)
Province, *n* (col %)				
Barcelona	3124 (90.2)	1491 (90.0)	1560 (90.4)	73 (90.1)
Lleida	183 (5.3)	86 (5.2)	93 (5.4)	4 (4.9)
Tarragona	95 (2.7)	40 (2.4)	52 (3.0)	3 (3.7)
Girona	60 (1.7)	39 (2.4)	20 (1.2)	1 (1.2)
Missing	2 (0.1)	-	2 (0.1)	-
Children under 17 years in the household, *n* (col %)				
One child	1279 (36.9)	623 (37.6)	641 (37.1)	15 (18.5)
Two children	1902 (54.9)	894 (54.0)	958 (55.5)	50 (61.7)
More than two children	283 (8.2)	139 (8.4)	128 (7.4)	16 (19.8)
Days since lockdown, mean (SD)	26.8 (3.0)	26.9 (3.5)	26.8 (3.5)	27.6 (4.2)

col %, column percent.

**Table 2:** Adherence to Mediterranean diet, and sleeping habits, after lockdown by gender.

	Total *n* = 3464	Girls *n* = 1656	Boys *n* = 1727	*p* Value
Adherence to Mediterranean diet, *n* (col %)				
Low (score < 4)	87 (2.5)	46 (2.8)	38 (2.2)	0.5165 ^a^
Medium (score 4–7)	1535 (44.3)	731 (44.1)	763 (44.2)	
High (Score ≥ 8)	1842 (53.2)	879 (53.1)	926 (53.6)	
KIDMED score, mean (SD)	7.5 (1.9)	7.5 (1.9)	7.5 (1.9)	0.4707 ^b^
Sleeping habits, *n* (col %)				
Delay bedtime	2745 (79.2)	1322 (79.8)	1360 (78.7)	0.4378 ^c^
Not adequate hours of sleep	666 (19.2)	295 (17.8)	349 (20.2)	0.0762 ^c^
DIMS score, mean (SD)	12.1 ± 4.3	12.2 ± 4.3	11.9 ± 4.3	**0.0038** ^b^
Suspected DIMS, n (col %)	563 (16.3)	286 (17.3)	263 (15.2)	0.1074 ^c^

*p* value was calculated by ^a^ Wilcoxson Mann–Whitney test, ^b^ T-test, or ^c^ chi-square test. DIMS, disorders of initiating and maintaining sleep. Bold numbers represent statistical significant values for an alpha level <0.05. col %, column percent.

**Table 3:** Adherence to Mediterranean diet and sleeping habits after lockdown by age range.

	<6 Years *n* = 1291 (37.3%)	6–10 Years *n* = 1263 (36.5%)	11–16 Years *n* = 910 (26.3%)	*p* Value
Adherence to Mediterranean diet, *n* (col %)				
Low (score < 4)	29 (2.3)	23 (1.8)	35 (3.8)	**<0.0001 ^a^**
Medium (score 4–7)	501 (38.8)	621 (49.2)	413 (45.4)	
High (Score ≥ 8)	761 (58.9)	619 (49.0)	462 (50.8)	
KIDMED score, mean (SD)	7.7 (1.9)	7.4 (1.9)	7.4 (2.1)	**<0.0001 ^b^**
Sleeping habits, *n* (col %)				
Delay bedtime,	881 (68.2)	1061 (84.0)	803 (88.2)	**<0.0001 ^c^**
Not adequate hours of sleep	257 (19.9)	360 (28.5)	49 (5.4)	**<0.0001 ^c^**
DIMS score, mean (SD)	12.9 (4.7)	11.8 (4.1)	11.3 (3.9)	**<0.0001 ^b^**
Suspected DIMS	273 (21.2)	183 (14.5)	107 (11.8)	**<0.0001 ^c^**

*p* value is calculated by ^a^ Kruskal–Wallis test, ^b^ one-way ANOVA, or ^c^ chi-square test. DIMS, disorders of initiating and maintaining sleep. Bold numbers represent statistical significant values for an alpha level <0.05. col %, column percent.

**Table 4:** Odds ratio of the inadequate frequency of physical activity associated with the implementation of the lockdown by gender and age range (total *n* = 3383 children).

	Total			<6 Years			6–10 Years Old			11–16 Years Old		
	OR	95% CI		OR	95% CI		OR	95% CI		OR	95% CI	
Girls *n* = 1656	1.8	1.5	2.1	1.2	0.9	1.6	2.2	1.6	3.1	2.4	1.7	3.4
Boys *n* = 1727	2.3	1.9	2.7	1.3	1.02	1.7	2.8	2.1	3.9	5.0	3.3	7.6

*p* value for the interaction among gender = 0.0617; *p* value for the interaction between the gender and age groups was <0.0001. Note: 81 individuals were excluded from this analysis due to missing gender.

**Table 5:** Odds ratio of the inadequate frequency of the use of TV-ED associated with the implementation of the lockdown by gender and age range (total *n* = 3383 children).

	Total			<6 Years			6–10 Years Old			11–16 Years Old		
	OR	95% CI		OR	95% CI		OR	95% CI		OR	95% CI	
Girls *n* = 1656	8.9	7.8	10.1	9.2	7.2	11.9	16.5	12.8	21.3	10.8	7.8	14.9
Boys *n* =1727	9.5	8.3	10.8	9.3	7.3	11.8	20.7	16.0	26.9	15.3	10.1	23.1

*p* value for the interaction among gender = 0.4563; interaction inter×gender×age group <0.05. Note: 81 individuals were excluded from this analysis due to missing gender.

## Data Availability

The corresponding author had full access to all the data in the study and data is available for the public upon request.

## Supplementary Materials

The following are available online at https://www.mdpi.com/article/10.3390/nu13051657/s1, Table S1: Demographic characteristics of included and excluded participants, Table S2: Affirmative answers in the KIDMED questionnaire, Figure S1: Physical activity and the use of TV-ED before and after lockdown by gender, Figure S2: Physical activity and the use of TV-ED before and after lockdown by age range, Figure S3: Physical activity alone or with relatives after lockdown, Figure S4: Mode of physical activity carried out after the lockdown,. Table S3: Recommendations for healthy habits during lockdown. Table S4: Parent’s perception of their children’s weight gain by levels of adherence to Mediterranean diet. Also available: Child Questionnaire.
