# Supplementary material for: Children’s Health Habits and COVID-19 Lockdown in Catalonia: Implications for Obesity and Non-Communicable Diseases
Source: Nutrients. 2021 May 13;13(5):1657. doi: 10.3390/nu13051657 (PMC8153273; doi:10.3390/nu13051657)
Supplement: Supplementary file 1 [file nutrients-13-01657-s001.zip › nutrients-1172155-supplementary.pdf]

## Supplementary tables and figures

**Table S1. Demographic characteristics of included and excluded participants.**

| | **Total *n* = 4704** | **Included *n* = 3464** | **Excluded *n* = 1240** |
|---|---|---|---|
| **Age range, *n* (col %)** | | | |
| < 6 years | 1846 (39.2%) | 1291 (37.13%) | 555 (44.8%) |
| 6 - 10 years | 1672 (35.5%) | 1263 (36.5%) | 409 (33.0%) |
| 11 - 16 years | 1186 (25.2%) | 910 (26.3%) | 276 (22.3%) |
| **Region, *n* (col %)** | | | |
| Catalonian Community | 3464 (73.6%) | 3464 (100%) | NA |
| Madrid Community | 497 (10.6%) | NA | 497 (40.1%) |
| Other | 743 (18.8%) | NA | 743 (59.9%) |
| **Children in the household, *n* (col %)** | | | |
| One | 1728 (36.7%) | 1279 (36.9%) | 449 (36.2%) |
| Two | 2540 (54.6%) | 1902 (54.9%) | 638 (51.5%) |
| More than two | 436 (9.3%) | 283 (8.2%) | 153 (12.3%) |
| **Days since lockdown, mean (SD)** | 26.9 (3.1) | 26.8 (3.0) | 27.1 (1.2) |

Ref: Col %: percentage by columns

**Table S2. Affirmative answers in the KIDMED questionnaire**

| | ***n* = 3464**<br>***n* (%)** |
|---|---|
| Consumes a fruit or fruit juice every day (+) | 2919 (84.3) |
| Has a second fruit every day (+) | 1609 (46.4) |
| Has Fresh or cooked vegetables regularly one per day (+) | 2628 (75.9) |
| Has Fresh or cooked vegetables more than once per day (+) | 1264 (36.5) |
| Consumes fish regularly (at least 2–3 per week) (+) | 2239 (64.6) |
| Goes to a fast-food restaurant more than once per week (-) | 0 (0) |
| Likes pulses and eats them more than once per week (+) | 2711 (78.3) |
| Consumes pasta or rice almost every day (5 or more times per week) (+) | 1530 (44.2) |
| Has cereal or cereal product for breakfast (+) | 2928 (84.5) |
| Consumes nuts regularly (at least 2–3/week) (+) | 1280 (37) |
| Uses of olive oil at home (+) | 3422 (98.8) |
| Skips breakfast (-) | 98 (2.8) |
| Has a dairy product for breakfast (+) | 3020 (87.2) |
| Has commercially baked goods or pastries for breakfast (-) | 956 (27.6) |
| Takes 2 yoghurts and/or 40 g cheese daily (+) | 1706 (49.2) |
| Takes sweets and candy several times a day (-) | 130 (3.8) |

Notes: Question (+) assigning a positive value of +1; question (-) assigning a (-) negative value of -1.

**Figure S1. Physical activity and the use of TV-ED before and after lockdown by gender.**

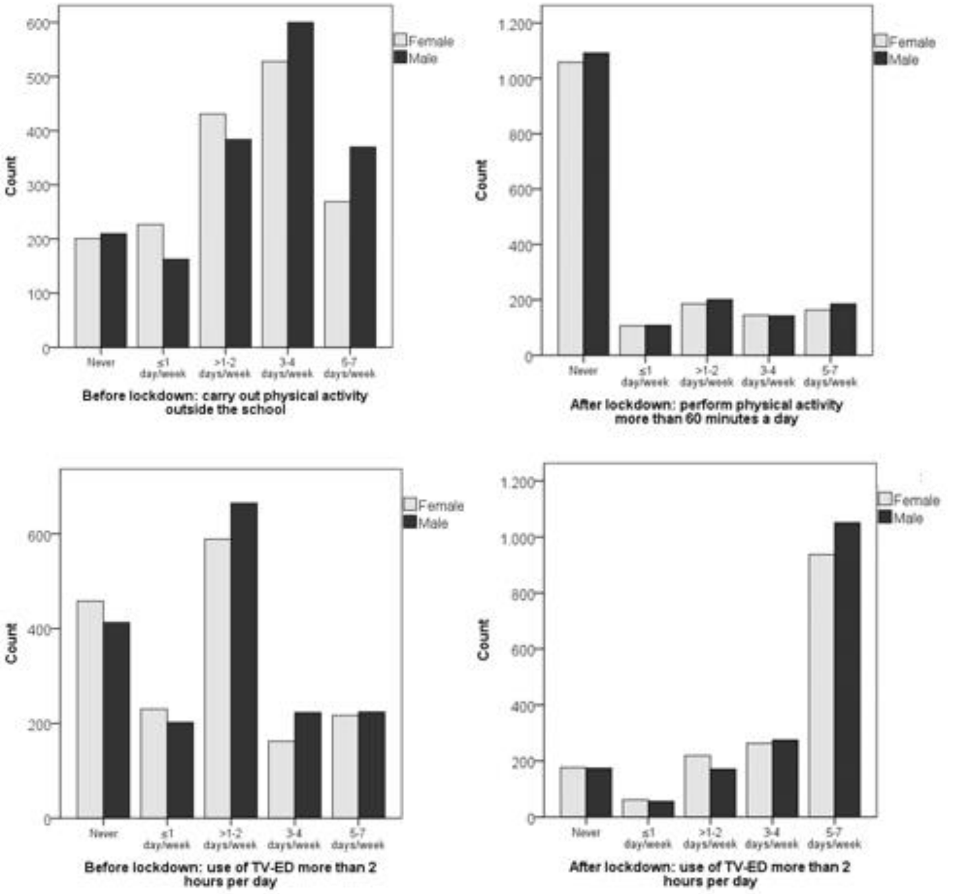


In the two upper figures, each column represents the frequency of days per week of carry out/perform physical activity 60 minutes a day before and after the lockdown by gender. In the two figures below, each column represents the frequency of days per week of use of TV-ED more than two hours per day before and after the lockdown by gender.

**Figure S2. Physical activity and the use of TV-ED before and after lockdown by age range**

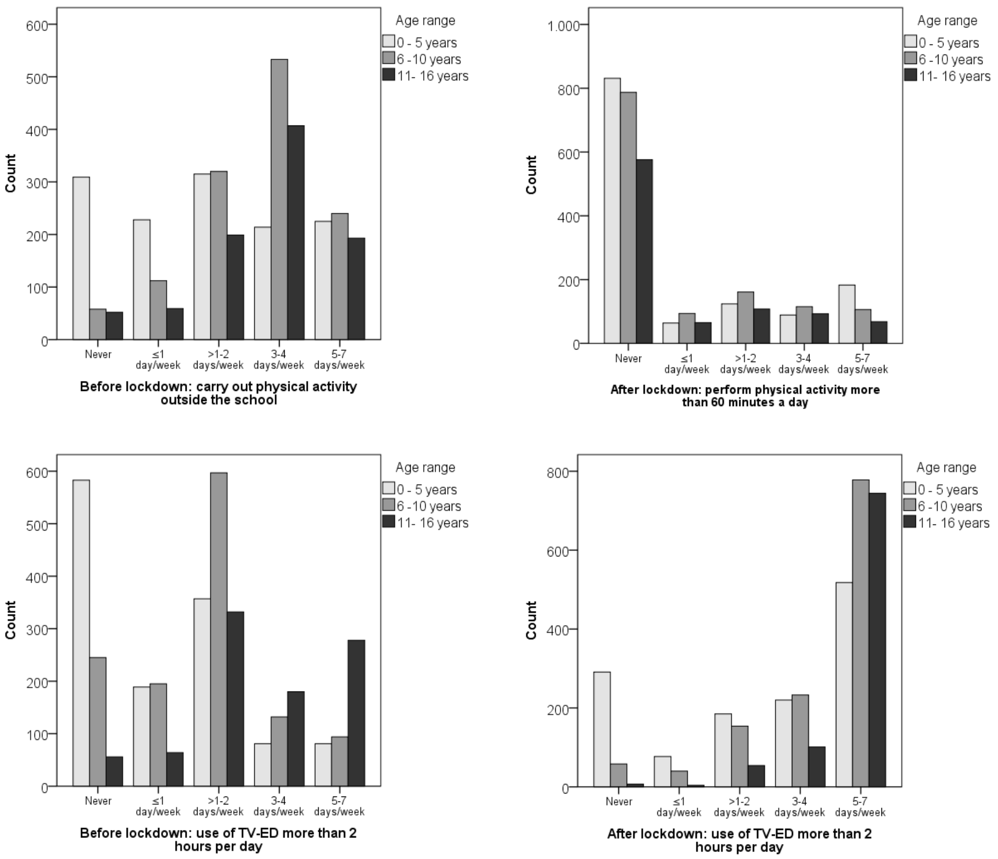


In the two upper figures, each column represents the frequency of days per week of carry out/perform physical activity 60 minutes a day before and after the lockdown by age range. In the two figures below, each column represents the frequency of days per week of use of TV-ED more than two hours per day before and after the lockdown by age range.

**Figure S3. Physical activity alone or with relatives after lockdown**

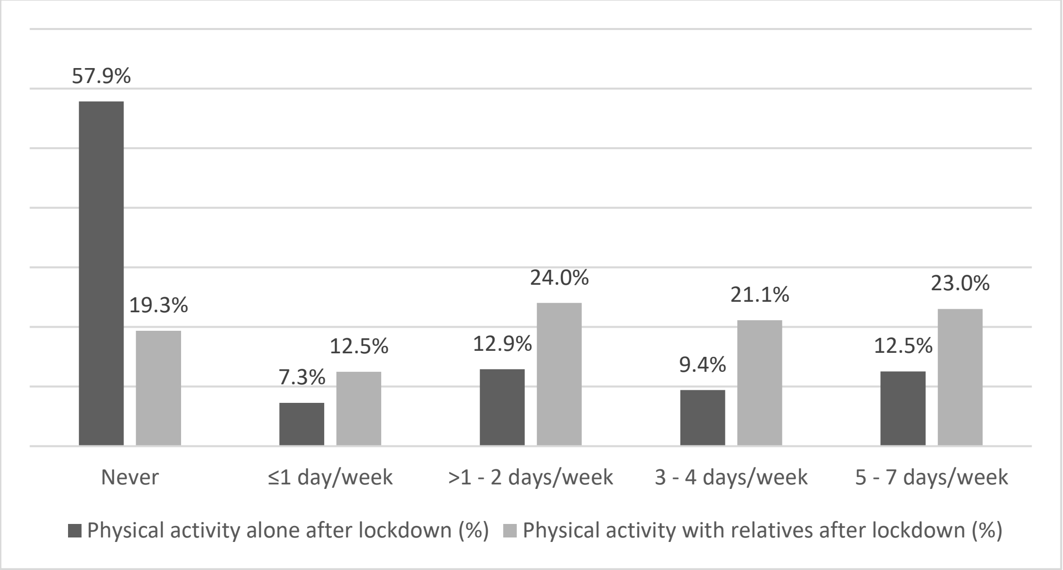


Each column represents the percentage of children in the sample that performed physical activity alone or with relatives after lockdown by weekly frequency

**Figure S4. Mode of physical activity carried out after the lockdown.**

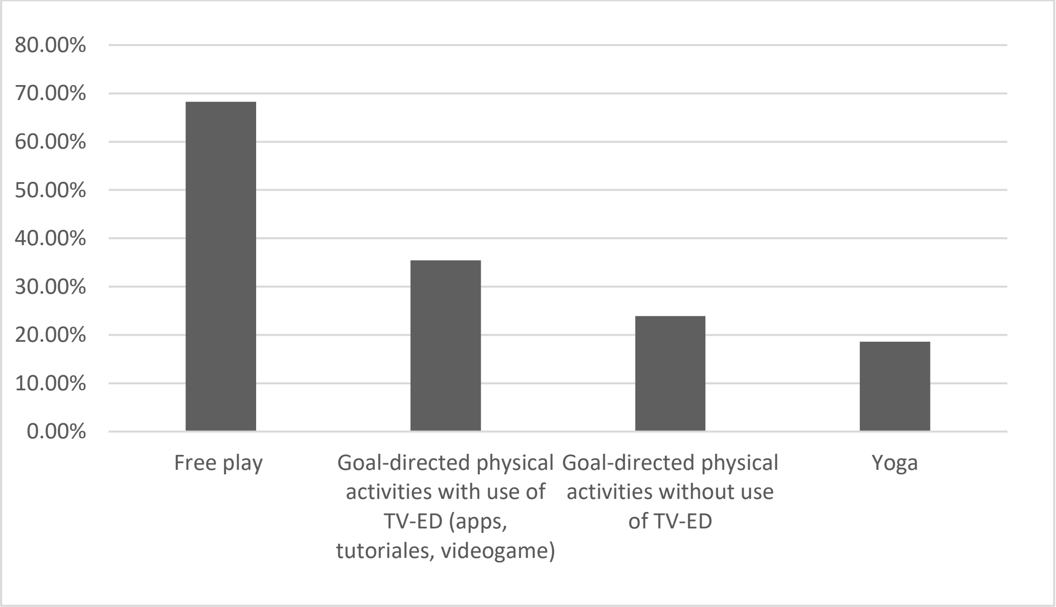


Each column represents the percentage kind of physical activity performed by the participants after the lockdown.

**Table S3. Recommendations for healthy habits during lockdown.**

| | Recommendation | Examples |
|---|---|---|
| **Diet** | **Stimulate social component of meals** | Avoid children eating in front of screens or eating alone [31,32,34].<br>Involve children in preparing food [32]. |
| | **Reduce chances of boredom and stress eating** | Increase frequency of meals but reduce the amount and calories of each meal. [31]<br>Increase portions of fruits and vegetables as snacks [31, 32] |
| | **Avoid stimulant food that could increase anxiety and disrupt sleeping** | Avoid beverages with caffeine content, particularly in the afternoon or before sleep time [33]. |
| **Sleeping** | **Maintain adequate circadian rhythm of sleep-wakefulness** | Try to have breakfast in a bright place [21].<br>Attach to same routine of wake up and bedtime over the weekdays [33,21].<br>Avoid use of TV-ED before sleeping [33, 34,21,36]. |
| | **Reduce stressful environment and anxiety** | Reduce COVID-19 information overload [21].<br>Allow children to share thoughts and feelings in a confident and non-judgmental environment [21].<br>Seek professional advice in case of severe anxiety. |
| | **Foster a relaxing environment for sleeping, preventing it from disrupting stimulus** | Avoid using bed for other activities than sleeping (not for watching movies, playing games, doing homework, or chatting) [21].<br>Establish pleasant environment in preparation to sleeping, like reading a book to children [33,21].<br>Reduce bright lights and disrupting noises in the bedroom particularly during sleeping hours [33].<br>Provide reassurance to reduce child anxiety or during awakenings rather than recurring to co-sleeping [21]. |
| **Physical activity** | **Incorporate physical activity into children's daily routine** | Plan breaks over the day to intercalate with sedentary activity, preferable in the morning or early in the afternoon, and maintain them over the weekdays (can be aligned to the time of the day that the use commuting) [5].<br>Plan different physical activities schedules for weekends. |
| | **Give priority to continuity and regularity over the intensity of the physical activity** | Organize short exercise routines that can be repeated during the day [5].<br>Increase difficulty or repetitions over weeks [11]. |
| | **Involve adults in guiding and sharing exercise** | Organize group activities that engage all family members in funny moments (dancing, group sports/games, board games that combine some physical activity) [36].<br>If there are children of different ages, consider also specific parental engagement for each age group. |
| **Use of TV-ED & sedentary behavior** | **Avoid prolonged exposures to screen or sedentary behavior** | Plan periods of screen exposure no longer than 1 hour [11].<br>Limit the duration of each sedentary activity, so as not to exceed 30-60 minutes [5, 36]. |
| | **Avoid 'sedentary' recreational screen use** | Engage in co-viewing and positive social interactions and experiences [36]. |
| | **Avoid using screen as mediator of other habits** | Discourage using screen for guiding physical activities unless there is no alternative and always under parental supervision [36].<br>If physical activity is followed through screen interaction, encourage family participation as well, by engaging in the activity or sharing the experience afterwards.<br>Avoid the use of screen during mealtimes or before sleeping [33].<br>Review multimedia messages together and encourage children to critically appraise them [37]. |
| | **Follow screen time use recommendations** | Place consistent limits on hours per day of media use as well as types of media used" (children 5+ years) [35]<br>Limit time screen to less than 60 min in young children (children 2–5 years) [34].<br>Switch screen time for play time wherever possible [35]. |

Note: Recommendations selected by the authors based on the study findings and guidelines released previous and during covid-19 pandemic [refs number]

**Table S4. Parent's perception of children's weight gain by levels of Adherence to Mediterranean Diet**

| **Adherence to Mediterranean diet** ***n*** **(col %)** | **Weight gain after the lockdown** | |
|---|---|---|
| | **No** | **Yes** |
| Optimal | 1444 (54.6%) | 398 (48.5%) |
| Suboptimal | 1199 (45.4%) | 423 (51.5%) |
| Total | 2643 | 821 |

Ref= Col %: percentage by columns
Each column represents the percentage of children who, according to the perception of their parents, gained (YES) or did not gain (NO) weight based on their adherence to the Mediterranean diet. The suboptimal adherence is the result of adding a low and medium adherence.

**Child Questionnaire**

**Study name:** Adherence to the Mediterranean diet, sleeping habits, and frequency of physical activity and use of TV and electronic devices during the lockdown period due to the COVID-19 pandemic in children and adolescents.

**Who we are and why are we releasing this survey?**
We are a multidisciplinary group of pediatric endocrinologists, nutritionists, sports doctors, and pediatricians from the HM Nens Hospital. We want to study the effects of the COVID-19 lockdown through a survey that will assess the nutrition, physical activity and sleep habits of children and adolescents.
We also want to give our support to families by providing healthy recipes.

**What are we expecting from you?**
We invite parents or responsible adult to complete this questionnaire on behalf of any child under 17 years of age living in the household. You need to answer a different questionnaire per each child under 17 age living in your household.
Completing each survey will only take about 5 minutes. This survey is completely anonymous and voluntary. At the end of the survey, we will direct you to a link on our website where you can download the recommendations for healthy recipes.

**This study has been approved by CEIm HM Hospitales (code 20.04.1585-GHM_COVID)**

**Thank you very much for collaborating! #staytathome#wecanmake it**

**Consent**

-I certified that I am older than 18 years of age and I consent to participate voluntarily in the study on "Adherence to the Mediterranean diet, physical activity and sleep during lockdown times due to the COVID-19 pandemic in children and adolescents" by completing the questionnaire on these habits of my daughter / child.

**Please complete with your own words the answer in the space given below each question.**

a. What country in the world are you in now?

---

b. What province do you live in?

---

c. What is your zip code?

---

d. How many children under the age of 17 do you have?

---

e. How many days has your child been in lockdown?

---

f. What is your child’s age group?
   - 0 to 5 years
   - 6 to 10 years
   - 11 to 16 years old

g. What is Your child's gender?
   - Woman
   - Man
   - I prefer not to say.

**Please answer the following questions about the habits of your child under 17 years of age DURING THE LOCKDOWN PERIOD**

Remember that each questionnaire corresponds to one child in the household (ONE questionnaire PER child)

You need to give just one option answer for each question unless it is specifically required in the question.

**I. Adherence to the Mediterranean diet DURING THE LOCKDOWN PERIOD**
[KIDMED questionnaire]

1. **Does your child…**
   a) …takes a fruit or fruit juice every day?
      - Yes
      - No
   b) …has a second fruit every day?
      - Yes
      - No
   c) …has fresh or cooked vegetables regularly once a day?
      - Yes
      - No

d) …has fresh or cooked vegetables more than once a day?
  - Yes
  - No

e) …consumes fish regularly (at least 2–3/week)?
  - Yes
  - No

f) Goes >1/ week to a fast-food restaurant (hamburger)?
  - Yes
  - No

g) Likes pulses and eats them >1/week?
  - Yes
  - No

h) Consumes pasta or rice almost every day (5 or more per week)?
  - Yes
  - No

i) Has cereals or grains (bread, etc) for breakfast?
  - Yes
  - No

j) Consumes nuts regularly (at least 2–3/week)?
  - Yes
  - No

k) Use olive oil at home?
  - Yes
  - No

l) Skips breakfast?
  - Yes
  - No

m) Has a dairy product for breakfast (yoghurt, milk, etc.)?
  - Yes
  - No

n) Has commercially baked goods or pastries for breakfast?
  - Yes
  - No

o) Takes two yoghurts and/or some cheese (40 g) daily?
  - Yes
  - No

p) Takes sweets and candy several times every day?
  - Yes
  - No

**2. In your opinion, has your child gained weight during these weeks of lockdown?**
  - Yes
  - No

## <u>II. Sleeping habits DURING THE LOCKDOWN PERIOD</u>

[The Sleep Disturbance Scale for Children (SDSC): disorders of initiating and maintaining sleep]

**1. Does your child go to sleep later than prior to lockdown?**
  - Yes

- No

2. **How many hours of sleep does your child get on most nights?**
    - 9 - 11 hours
    - 8 - 9 hours
    - 7 - 8 hours
    - 5 - 7 hours
    - Less than 5 hours

3. **How long after going to bed does your child usually fall asleep?**
    - Less than 15 minutes
    - 15 - 30 minutes
    - 30 - 45 minutes
    - 45 - 60 minutes
    - More than 60 minutes

4. **Does your child go to bed reluctantly?**
    - Never
    - Occasionally (once or twice per month or less)
    - Sometimes (once or twice per week)
    - Often (3-5 times per week)
    - Always (daily)

5. **Has your child any difficulty getting to sleep at night?**
    - Never
    - Occasionally (once or twice per month or less)
    - Sometimes (once or twice per week)
    - Often (3-5 times per week)
    - Always (daily)

6. **Does your child feel anxious or afraid when falling asleep?**
    - Never
    - Occasionally (once or twice per month or less)
    - Sometimes (once or twice per week)
    - Often (3-5 times per week)
    - Always (daily)

7. **Does your child wake up more than twice per night?**
    - Never
    - Occasionally (once or twice per month or less)
    - Sometimes (once or twice per week)
    - Often (3-5 times per week)
    - Always (daily)

8. **Does your child has difficulty to fall asleep again after waking up in the night?**
    - Never
    - Occasionally (once or twice per month or less)
    - Sometimes (once or twice per week)
    - Often (3-5 times per week)
    - Always (daily)

**Please answer the following questions about the habits of your child had in a typical day or week BEFORE THE LOCKDOWN PERIOD and the habits your child currently has DURING THE LOCKDOWN PERIOD**

Remember that each questionnaire corresponds to one child in the household (ONE questionnaire PER child)

You need to give just one option answer for each question unless it is specifically required in the question.

**III. Physical activity questionnaire** [Adapted from WHO physical activity guidelines]

1. **Under normal conditions (without lockdown), how many days a week does your child engage in PHYSICAL activity outside of school?**
    - Never
    - 1 day a week
    - 1 to 2 days a week
    - 3 to 4 days a week
    - Every or almost every day of the week

2. **During lockdown how many days a week does your child exercise between 30 and 60 minutes a day?**
    - Never
    - 1 day a week
    - 1 to 2 days a week
    - 3 to 4 days a week
    - Every or almost every day of the week

3. **During lockdown how many days a week does your child exercise more than 60 minutes a day?**
    - Never
    - 1 day a week
    - 1 to 2 days a week
    - 3 to 4 days a week
    - Every or almost every day of the week

4. **During lockdown how many days a week does your child exercise alone?**
    - Never
    - 1 day a week
    - 1 to 2 days a week
    - 3 to 4 days a week
    - Every or almost every day of the week

5. **During lockdown how many days a week does your child exercise with other family members?**
    - Never
    - 1 day a week

- 1 to 2 days a week
- 3 to 4 days a week
- Every or almost every day of the week

6. **Indicate the sport (s) that your child does <u>during lockdown</u> (more than one option is possible)**
    - Free play
    - Directed series circuit
    - Video game-guided physical activity
    - Dance
    - Yoga
    - Mobile apps or internet tutorials
    - Static machines
    - None
    - Other

**<u>IV. Use of TV and electronic devices.</u>** [Adapted from WHO guidelines for TV use]

1. **Under <u>normal conditions</u> (without lockdown), how many days a week does your child use television, computer (not academic) or mobile for MORE than 2 hours a day?**
    - Never
    - 1 day a week
    - 1 to 2 days a week
    - 3 to 4 days a week
    - Every or almost every day of the week
2. **<u>During lockdown</u> how many days a week does your child use television, computer (not academic) or mobile phone for MORE than 2 hours in a day?**
    - Never
    - 1 day a week
    - 1 to 2 days a week
    - 3 to 4 days a week
    - Every or almost every day of the week

**You reached the end of the questionnaire.**

**What else you need to know?**

<u>Personal data protection</u>

The data provided will be treated in accordance with Organic Law 3/2018, of December 5, on the Protection of Personal Data and guarantee of digital rights and will follow national and international guidelines (code of ethics, Declaration of Helsinki). All information obtained will be treated confidentially, in accordance with the new General Data Protection Regulation (RGPD): "Regulation (EU) 2016/679 of the European Parliament and of the Council of April 27, 2016 on Data Protection (RGPD) ".

- I accept.
- I do not accept.

**Thank you for your time! Check our website for healthy recipes and recommendations during lockdown at**
https://hospitaldenens.com/es/destacados/estudio-covid19/

**References**

[8, 9,11]

Serra-Majem L, Ribas L, Ngo Jet al. Food, youth and the Mediterranean diet in Spain. Development of KIDMED, Mediterranean Diet Quality Index in children and adolescents. Public Health Nutr. 2004;7(7):931–5.

Bruni O, Ottaviano S, Guidetti Vet al. The Sleep Disturbance Scale for Children (SDSC) Construct ion and validation of an instrument to evaluate sleep disturbances in childhood and adolescence. J Sleep Res. 1996;5(4):251–61.

WHO guidelines on physical activity and sedentary behaviour [Internet]. [cited 2021 Jan 6]. Available from: https://www.who.int/publications/i/item/9789240015128
